# Explorations of predictors for parametrial invasion and how it affects treatment strategy for bulky cervical cancer

**DOI:** 10.3389/fonc.2025.1660495

**Published:** 2025-09-30

**Authors:** Jing Yang, Jianan Ji, Xiao Ni, Yang Li, Ting Chen, Jing Zhang, Jiangnan Qiu, Chengyan Luo

**Affiliations:** ^1^ Department of Gynecology, First Affiliated Hospital with Nanjing Medical University, Nanjing, China; ^2^ Department of Pathology, First Affiliated Hospital with Nanjing Medical University, Nanjing, China; ^3^ Department of Radiology, First Affiliated Hospital with Nanjing Medical University, Nanjing, China

**Keywords:** bulky cervical cancer, parametrial invasion, radical surgery and adjuvant radiotherapy, concurrent chemoradiotherapy, prognosis

## Abstract

**Objectives:**

The treatment of bulky cervical cancer (BCC) remains challenging owing to poor local control and susceptibility to distant metastasis. The study aimed to identify the factors that affect the development of parametrial invasion (PMI) and evaluate their association with different treatment modalities for BCC.

**Methods:**

The retrospective study enrolled 462 women with BCC treated at our center between January 2010 and June 2023. Logistic regression was utilized to analyze the factors influencing PMI. Kaplan-Meier method and Cox proportional hazard model were employed for survival analyses.

**Results:**

FIGO stage III-IV and primary tumor volume > 16 cm³ were identified as independent predictors for PMI in BCC, with ORs of 6.45 (95% CI 2.43-18.01) and 2.11 (95% CI 1.25-3.61), respectively. Multivariate Cox regression analysis demonstrated that the presence of PMI was associated with poorer progression-free survival (PFS) (HR 1.72, 95% CI 1.02-2.9), but exerted no significant effect on cancer-specific survival (CSS) (HR 0.86, 95% CI 0.37-2.0). The patients with FIGO stage I-II disease and no PMI who received radical surgery and adjuvant radiotherapy (RS and ART) presented improved PFS than those receiving concurrent chemoradiotherapy (CCRT), whereas CSS was unaffected. The cases at FIGO stage I-II with PMI exhibited no significant difference in PFS or CSS between receiving different treatments. No discernible difference in the incidence of grade ≥ 3 radiotherapy-related adverse events and quality of life was observed between treatment groups at 3- and 6-month post-treatment.

**Conclusions:**

BCC patients with FIGO stage III-IV and tumor volume > 16 cm^3^ were more susceptible to PMI. RS and ART improved PFS in patients with FIGO stage I-II disease and no PMI without increasing risks for serious adverse events and impairing the patients’ quality of life.

## Introduction

Uterine cervical cancer (UCC) ranks fourth in terms of incidence and mortality rates among female malignancies worldwide, with an estimated 660,000 new cases and 350,000 deaths in 2022 (1). In China, despite the nationwide cervical cancer screening program and HPV vaccination programs, the incidence and mortality rates of cervical cancer have not shown a remarkable decline, with 150,700 new cases and 55,700 deaths in 2022 (2). Bulky cervical cancer (BCC), also known as locally advanced cervical cancer (LACC), is defined as cervical malignancies staged at International Federation of Obstetrics and Gynecology (FIGO) 2018 stage IB3, IIA2, and above with a primary tumor size greater than 4cm. According to a systematic review by Monk et al. (3) that included 40 studies from 28 countries worldwide, LACC was estimated to accounts for 37% of all UCC cases (interquartile range [IQR] 25.8, 52.1). Previous studies have confirmed that BCC tends to develop deep interstitial infiltration and lymph node metastasis, with 5-year overall survival (OS) and progression-free survival (PFS) rates of about 70% and 60%, respectively (4, 5). According to the National Comprehensive Cancer Network (NCCN) guideline, platinum-based concurrent chemoradiotherapy (CCRT) is recommended for stage IB3 and IIA2 (category 1), while radical hysterectomy (RH) with pelvic lymph node dissection (PLND) with or without para-aortic lymph node dissection (PALD) is also an optional regimen (category 2B). For LACC with parametrial invasion (PMI), CCRT remains the standard treatment (6). Despite the availability of advanced technologies including intensity-modulated radiotherapy (IMRT), image-guided adaptive brachytherapy (IGAB) along with interstitial technique, the treatment of BCC remains a concern owing to the large volume of primary tumor with poor local control to CCRT and susceptibility to distant metastasis (7–11). Besides, radiotherapy may result in adverse reactions, including radiation enteritis, cystitis and vaginitis, which adversely affect the patients’ quality of life.

In China, a subset of clinicians prefers radical surgery (RS) to CCRT for BCC patients at FIGO stage IB3, IIA2, and IIIC without parametrial invasion (PMI). This preference mainly stems from two factors: the availability to radiotherapy that varies across regions in China, and socio-cultural considerations that impact the patients’ willingness for surgical resection (12, 13). Therefore, determining the presence (or absence) for PMI in BCC plays an important role in clinical decision making, which relies on gynecologic examination and pelvic magnetic resonance imaging (MRI). However, the diagnostic accuracy for PMI detection significantly decreases as the tumor diameter increases (14). To date, limitations have been noted in the various assays for determining whether BCC is concomitant with PMI. It should be acknowledged that the large dimensions of the local tumor, its closeness to surrounding organs, the narrowness of the paracervical space, and the abundance of vessels complicate the surgical procedure and lead to higher risk of complications, including intra-operative hemorrhage and injuries to the ureters and bladders (15, 16). Therefore, the treatment of BCC remains challenging, necessitating the optimized individualized strategies. This study aimed to investigate the risk factors for PMI in patients with BCC and the impact of receiving different treatments on oncologic outcomes and quality of life, hoping to provide evidence for optimal BCC treatment strategies.

## Materials and methods

### Study design and data extraction

Patients with pathologically confirmed UCC treated at our center between January 2010 and June 2023 were enrolled in this study and retrospectively analyzed. The pre-treatment MRI scans before treatment were reviewed by two senior radiologists. All tissue sections were evaluated by one senior pathologist. The inclusion criteria were: (1) pathologically confirmed primary UCC, (2) having received initial treatment, (3) tumors larger than 4cm on pre-treatment pelvic MRI, and (4) a minimum follow-up of 3 months. The exclusion criteria were: (1) diagnosis of other malignancies within the preceding 3 months, (2) metastatic cervical tumors, (3) incomplete clinical data, (4) prior pelvic radiation treatment, (5) loss of follow-up, and (6) survival duration < 3 months. We collected clinicopathological information by reviewing the electronic medical records. The greatest tumor diameter and tumor volume were assessed via pelvic MRI, with the tumor volume computed using the formula: length * breadth * width * 0.52 (cm³). PMI was determined by two gynecologic oncologists through pelvic examination or by two senior radiologists who reviewed the films together. In case of disagreement, the less severe assessment was adopted. The continuous variables including age, largest tumor diameter, and tumor volume were thresholded using X-tile software (17) (https://medicine.yale.edu/lab/rimm/research/software/) and converted to dichotomous variables with cutoff values of 54 years, 4.8cm, and 16 cm^3^. The FIGO 2018 staging system was employed in the study. Owing to the limited number of FIGO stage IV cases (only 8), the cohort was classified into two groups: FIGO stage I-II and FIGO stage III-IV. These patients were treated with either CCRT and vaginal brachytherapy, or radical surgery and adjuvant radiotherapy (RS and ART).

**Figure 1:**
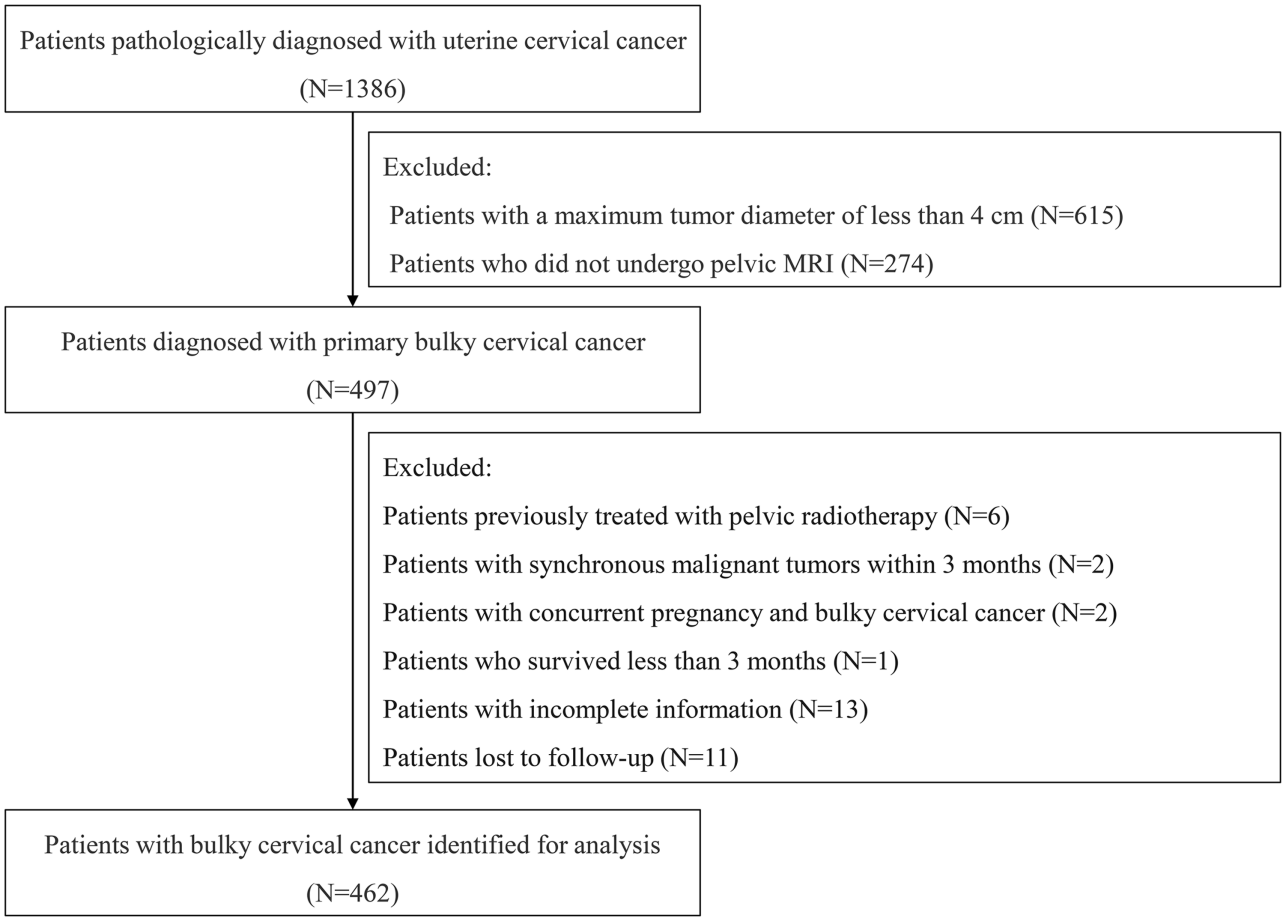
Flowchart of data processing for this study.

We evaluated the patients’ quality of life by utilizing The European Organization for Research and Treatment of Cancer, Quality of Life Questionnaire Cervical Cancer Module (EORTC QLQ-CX24) (18) at 3 months and 6 months post-treatment. Surgery- and radiotherapy-related complications were also assessed using the Clavien-Dindo (CD) grading system (19) and the Radiation Therapy Oncology Group (RTOG) grading system (20), respectively. The patients were followed up by reviewing the outpatient medical records and by telephone interview. Cancer-specific survival (CSS) is defined as the interval from diagnosis to either death attributable to UCC, or to the last follow-up for the surviving patients. PFS was defined as the interval from diagnosis to either tumor progression, or death, or to the last follow-up for those without progression. The flowchart of data processing is shown in **Figure 1**. The study was conducted in accordance with the Declaration of Helsinki and was reviewed and approved by the Ethics Committee of the First Affiliated Hospital with Nanjing Medical University (No. 2024-SR-261). Informed consent was obtained from all participants.

### Statistical analyses

In this study, data were collected using Excel 2010 and Epidata 3.1 software (https://epidata.dk/download.php) and analyzed by using Statistical Product and Service Solutions (SPSS) software 26.0 (IBM Corps., Armonk, NY, USA) and R software (https://www.r-project.org/, version 4.1.3). Quantitative data that followed a normal distribution were reported as mean ± standard deviation (SD), while data that deviated from normality were reported as median (IQR). The t-test and Mann-Whitney test were utilized to compare differences between the groups accordingly. Qualitative data was expressed in terms of numbers (percentages), with the chi-square test applied to evaluate differences. Logistic regression was utilized to analyze the factors associated with PMI, and Cox proportional hazard model was employed to investigate the predictive factors affecting the prognosis in BCC. PFS and CSS were plotted by using the Kaplan-Meier method, and the Log-rank test was applied to clarify the differences between the groups. Differences were considered statistically significant when P < 0.05.

## Results

### Baseline characteristics of the population

A total of 462 women with BCC treated at our center from January 2010 to June 2023 were included in this study, with a median age of 47 years (IQR 42, 54) and a median body mass index (BMI) of 23.45 (IQR 21.6, 25.6). Of these cases, 372 (80.52%) were squamous carcinomas, 72 (15.58%) were adenocarcinomas, 10 (2.16%) were adenosquamous carcinomas, 6 (1.30%) were neuroendocrine carcinomas, and 2 (0.43%) were carcinosarcomas. There were 211 cases (45.67%) at FIGO stage I, 148 cases (32.03%) at stage II, 95 cases (20.56%) at stage III, and 8 cases (1.73%) at stage IV. Based on the pre-treatment MRI findings, the median tumor diameter was 4.50cm (IQR 4.03, 5.00) and the median tumor volume was 14.35 cm^3^ (IQR 7.89, 25.50). By means of combined gynecological examination and pelvic MRI, 91 patients (19.70%) were identified with PMI and 371 (80.30%) without PMI. 384 patients (83.12%) underwent RS and ART, whereas 78 patients (16.88%) were treated with CCRT. The baseline characteristics are summarized in **Table 1**. Up to December 2023, the median follow-up was 65 months (IQR 38, 84.5), the 3- and 5-year PFS rates were 84.2% and 78.2%, and the 3- and 5-year CSS rates were 95.3% and 93%, respectively.

**Table 1:** Clinical and pathological characteristics of 462 patients with BCC.

Characteristics	Number (%)
Age, years ^†^	47 (42, 54)
BMI, kg/m^2 †^	23.4 (21.6, 25.5)
Menopause	
No	289 (63)
Yes	173 (37)
FIGO stage	
I and II	359 (78)
III and IV	103 (22)
Diameter, cm	
≤ 4.8	276 (60)
> 4.8	186 (40)
Volume, cm^3^	
≤ 16	250 (54)
> 16	212 (46)
PMI	
No	371 (80)
Yes	91 (20)
Histological grade	
I	24 (5.2)
II	202 (44)
III-IV	236 (51)
Histology type	
SCC	372 (81)
AC	72 (16)
Others	18 (3.9)
LVSI	
No	372 (81)
Yes	198 (43)
Depth	
Superficial 1/3	70 (15)
Mediate 1/3	136 (29)
Deep 1/3	256 (55)
LNM	
No	378 (82)
Yes	84 (18)
Treatment	
RS and ART	384 (83)
CCRT	78 (17)

^†^The values are given as the median (IQR, interquartile range). AC, adenocarcinoma; BCC, bulky cervical cancer; BMI, body mass index; CCRT, concurrent chemoradiotherapy; FIGO, International Federation of Obstetrics and Gynecology; LNM, lymph node metastasis; LVSI, lymph-vascular space invasion; PMI, parametrial invasion; RS and ART, radical surgery and adjuvant radiotherapy; SCC, squamous carcinoma.

### Factors determining the occurrence of PMI and its impact on the prognosis of patients with BCC

**Figure 2:**
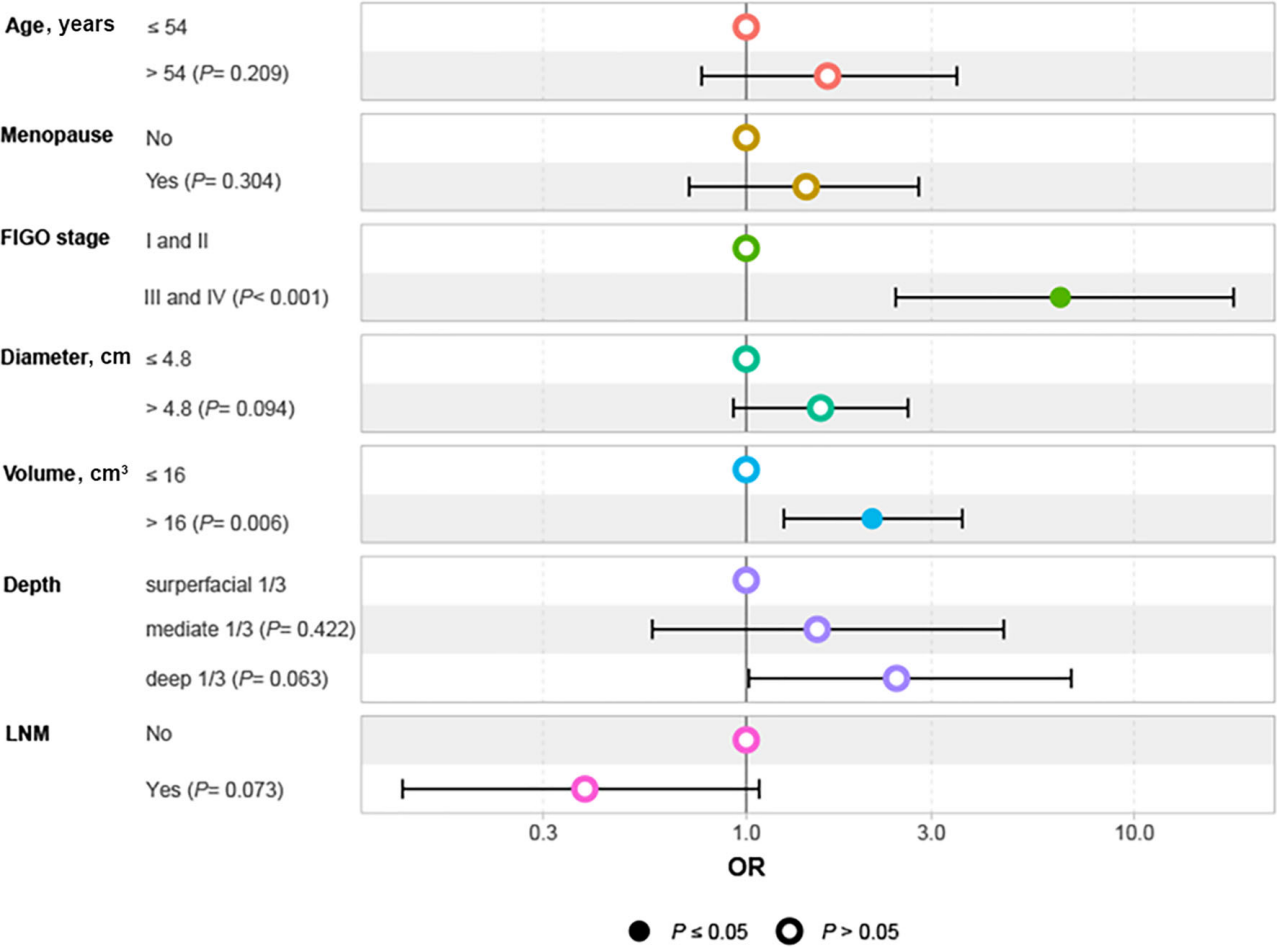
Forest plot of independent factors affecting PMI in patients with BCC. BCC, bulky cervical cancer; FIGO, International Federation of Obstetrics and Gynecology; LNM, lymph node metastasis; OR, odds ratio; PMI, parametrial invasion.

**Figure 3:**
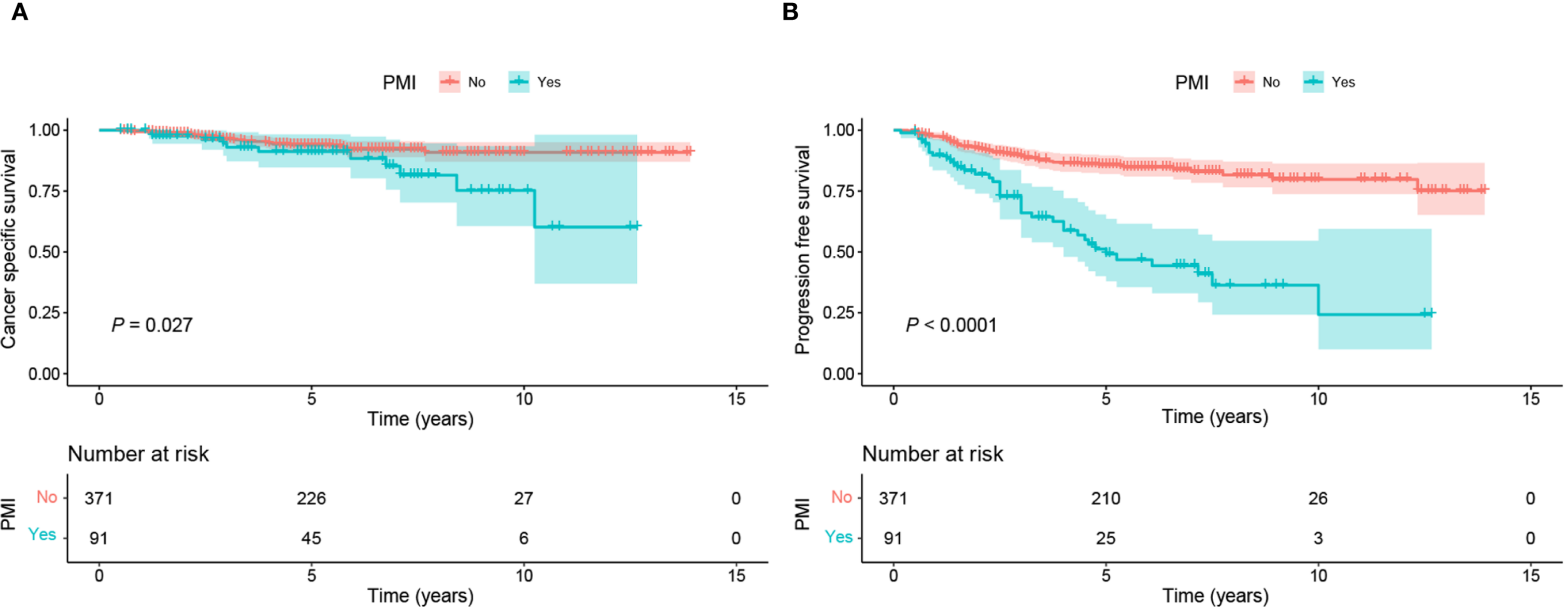
Kaplan-Meier curves of CSS and PFS in BCC patients with and without PMI. **(A)** Patients without PMI had superior CSS to those with PMI (P=0.027). **(B)** Patients without PMI had superior PFS to those with PMI (P <0.0001). BCC, bulky cervical cancer; CSS, cancer-specific survival; PFS: progression-free survival; PMI, parametrial invasion.

Given that the accuracy of PMI evaluation may be compromised by the crushing effect from the big local tumor and inflammatory edema with the parametrial tissues, we performed logistic regression analysis to identify the factors associated with PMI in BCC. Univariate and multivariate logistic regression analyses revealed that FIGO stage III-IV (OR 6.45, 95%CI 2.43-18.01, P < 0.001) and tumor volume >16 cm³ (OR 2.11, 95%CI 1.25-3.61, P=0.006) were independent risk factors for the occurrence of PMI in BCC (**Table 2**). The findings are visualized using a forest plot (**Figure 2**). Additionally, we examined the prognostic significance of PMI on the prognosis in BCC cases and discovered that individuals with PMI exhibited poorer CSS and PFS compared to those without PMI (**Figure 3**).

**Table 2:** Univariate and multivariate logistic regression analysis to determine predictors for PMI in 462 patients with BCC.

Variable	Univariate		Multivariate	
	OR (95%CI)	P value	OR (95%CI)	P value
Age, years				
≤ 54	Reference		–	
> 54	1.7 (1.02-2.83)	0.04^*^	1.62 (0.77-3.49)	0.21
Menopause				
No	Reference		-	
Yes	1.66 (1.04-2.61)	0.03^*^	1.43 (0.71-2.78)	0.30
BMI, kg/m^2^				
≤ 25	Reference			
> 25	0.66(0.4-1.11)	0.12		
FIGO stage				
I and II	Reference		-	
III and IV	3.83 (2.34-6.29)	< 0.001^***^	6.45 (2.43-18.01)	< 0.001^***^
Depth				
Superficial 1/3	Reference		–	
Mediate 1/3	1.63 (0.62-4.3)	0.33	1.52 (0.57-4.61)	0.42
Deep 1/3	3.78 (1.57-9.13)	0.003^**^	2.44 (1.02-6.88)	0.06
Diameter, cm				
≤ 4.8	Reference		–	
> 4.8	2.11 (1.33-3.35)	0.002^**^	1.55 (0.93-2.61)	0.09
Volume, cm^3^				
≤ 16	Reference		-	
> 16	2.79 (1.72-4.51)	< 0.001^***^	2.11 (1.25- 3.61)	0.006^**^
LNM				
No	Reference		–	
Yes	2.5 (1.47-4.24)	0.001^**^	0.38 (0.13-1.08)	0.07
Histological grade				
I	Reference			
II	0.69 (0.24-1.98)	0.49		
III-IV	1.15 (0.41-3.24)	0.78		
Histology type				
SCC	Reference			
AC	0.9 (0.47-1.73)	0.76		
Others	1.58 (0.54-4.56)	0.40		
LVSI				
No	Reference			
Yes	0.95 (0.59-1.5)	0.81		

Bold values are of statistical significance. *P values < 0.05; **P values < 0.01; ***P values < 0.001. AC, adenocarcinoma; BCC, bulky cervical cancer; BMI, body mass index; CI, confidence interval; FIGO, International Federation of Obstetrics and Gynecology; LNM, lymph node metastasis; LVSI, lymph-vascular space invasion; OR, odds ratio; PMI, parametrial invasion; SCC, squamous carcinoma.

### Factors affecting the prognosis of BCC

**Figure 4:**
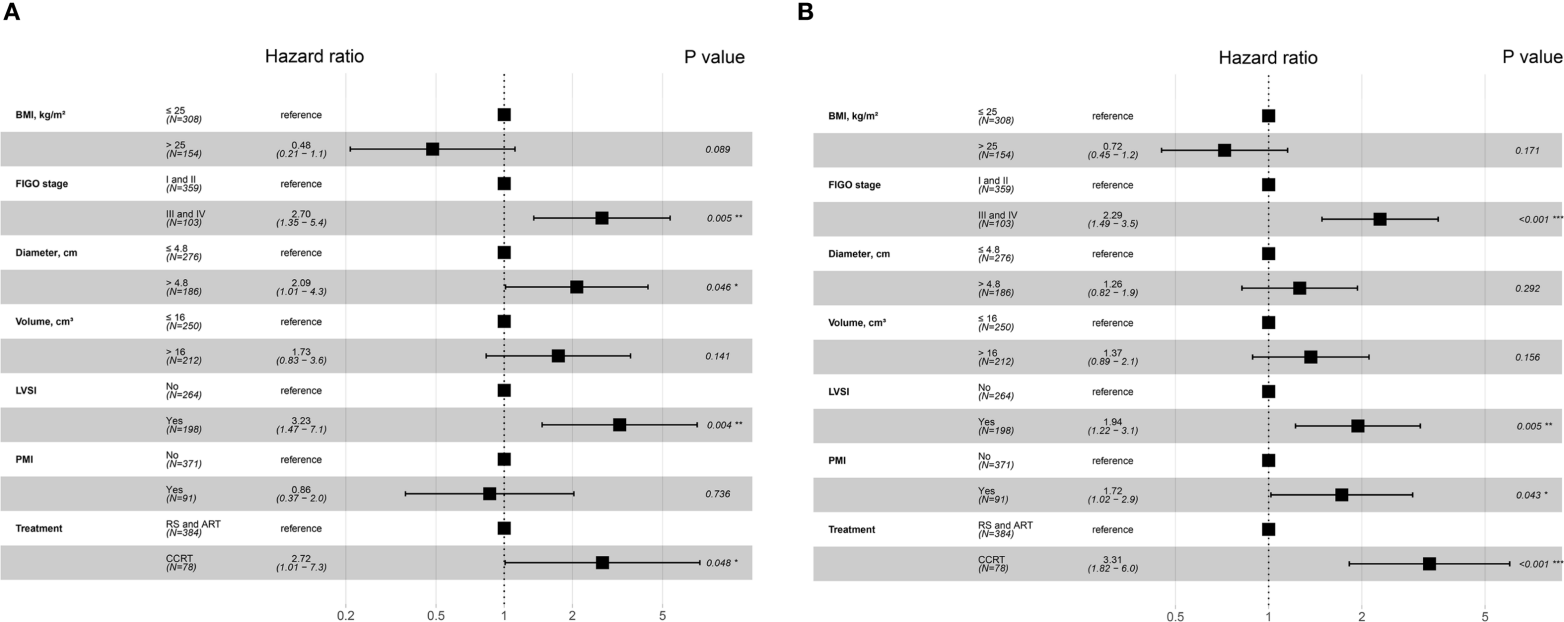
Forest plot of multivariate Cox regression analysis for independent factors associated with CSS and PFS in BCC patients. **(A)** Factors for predicting CSS in patients with BCC. **(B)** Factors for predicting PFS in patients with BCC. *P values < 0.05; **P values < 0.01; ***P values < 0.001. BCC, bulky cervical cancer; BMI, body mass index; CCRT, concurrent chemoradiotherapy; CSS, cancer-specific survival; FIGO, International Federation of Obstetrics and Gynecology; LVSI, lymph-vascular space invasion; PFS, progression-free survival; PMI, parametrial invasion; RS and ART, radical surgery and adjuvant radiotherapy.

The study initially established that the BCC patients with PMI presented a worse prognosis. To determine whether this association was influenced by other variables, we conducted Cox regression analysis. We verified that the Cox model satisfied the proportional hazards assumption and assessed the multicollinearity between the variables. Multicollinearity between FIGO stage and lymph node (LN) metastasis was observed when CSS was employed as the outcome (variance inflation factor [vif] 5.10), and between FIGO stage and depth of mesenchymal invasion when PFS was employed as the outcome (vif 5.7). Accordingly, LN metastasis and depth of mesenchymal invasion were excluded in the subsequent analyses. Univariate Cox regression analysis revealed that patients receiving CCRT, and those with BMI ≤ 25, FIGO stage III-IV, presence of PMI, tumor diameter > 4.8cm, and tumor volume > 16 cm³ had shorter CSS and PFS. In terms of CSS, in addition to the factors outlined above, the cases with LVSI had a higher risk of mortality. Multivariate Cox regression analysis further demonstrated that FIGO stage III-IV (HR 2.7, 95% CI 1.35-5.4), tumor diameter > 4.8cm (HR 2.09, 95% CI 1.01-4.3), the presence of LVSI (HR 3.23, 95% CI 1.47-7.1), and receiving CCRT were associated with an increased risk of tumor-specific deaths in BCC patients (**Figure 4A**). FIGO stage III-IV (HR 2.29, 95% CI 1.49-3.5), the presence of LVSI (HR 1.94, 95% CI 1.22-3.1) and PMI (HR 1.72, 95% CI 1.02-2.9), and undergoing CCRT (HR 3.31, 95% CI 1.82-6.0) were independent prognostic factors for PFS (**Figure 4B**). These findings demonstrate that PMI was an independent predictor of PFS, but not CSS, for BCC.

### Effects of different treatments on oncologic outcomes in BCC patients at early stage

**Figure 5:**
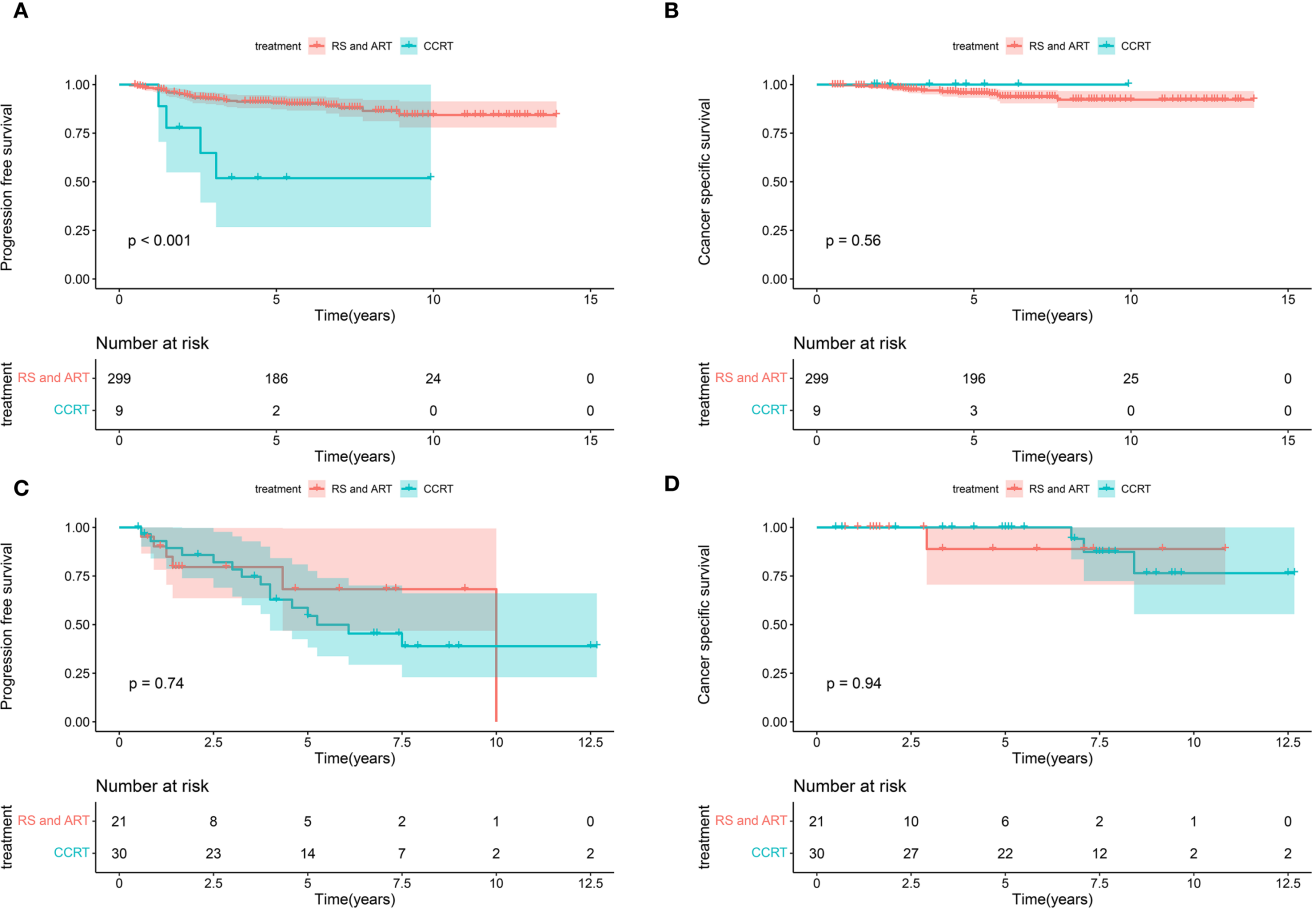
Kaplan-Meier curves of CSS and PFS in patients with BCC at early stage receiving different treatments. **(A)** The patients with FIGO stage I-II disease and no PMI who received RS and ART had improved PFS than those who received CCRT (P < 0.001). **(B)** The patients with FIGO stage I-II disease and no PMI presented no significant difference in terms of CSS between receiving RS and ART and CCRT (P=0.56). **(C)** The women at FIGO stage I-II with PMI exhibited no significant difference in PFS between receiving RS and ART and CCRT (P=0.74). **(D)** The women at FIGO stage I-II with PMI exhibited no significant difference in CSS between receiving RS and ART and CCRT (P=0.94). CSS, cancer-specific survival; PFS, progression-free survival; BCC, bulky cervical cancer; FIGO, International Federation of Obstetrics and Gynecology; PMI, parametrial invasion; RS and ART, radical surgery and adjuvant radiotherapy; CCRT, concurrent chemoradiotherapy.

The treatment of BCC presents challenges for clinicians. Our findings showed that individuals with PMI exhibited poorer PFS compared to those without PMI. In clinical practice, PMI is an essential factor for patients with UCC when deciding between surgery and CCRT, particularly in the early stages. The prognosis of BCC patients at FIGO stage I-II was further analyzed in conjunction with PMI and various treatments. The results showed that patients with FIGO stage I-II disease and no PMI who received RS and ART had improved PFS than those who received CCRT, whereas CSS was unaffected (**Figures 5A, B**). The cases at FIGO stage I-II with PMI exhibited no significant difference in PFS or CSS between those receiving RS and ART and those receiving CCRT (**Figures 5C, D**). The above results indicate that RS and ART can only improve PFS in BCC patients without PMI at early-stage, but have no effect on CSS. Furthermore, they fail to enhance PFS or CSS in those with PMI.

### Effects of different treatments on quality of life in patients with BCC

We also assessed the quality of life of all BCC patients at 3- and 6- months post-treatment for all BCC patients using the EORTC QLQ-CX24. No significant difference was found in the quality-of-life scores of patients who underwent RS and ART versus CCRT. At 3 months, the scores were 29.25 ± 4.38 in RS and ART group versus 27.00 ± 3.13 in CCRT group (P=0.108), and at 6 months, the scores were 22.75 ± 2.77 and 23.16 ± 2.65 (P=0.689), respectively (**Table 3**). Regarding treatment complications, 9 (2.3%) of the 384 patients who underwent RS and ART had class CD grade 3 surgery-related complications. Among them, 6 cases received intraoperative ureteral stents placement to prevent ureteral fistulas, due to large local tumors and poor ureteral blood supply after cutting off the parametrial tissue. 3 cases had ureteral stents placed for severe hydronephrosis at 6 months postoperatively. All affected patients maintained normal renal function during follow-up. There was no significant difference (P=0.293) between RS and ART versus CCRT in terms of grade ≥ 3 radiologic damage, with 21 (5.5%) and 7 (9.0%) patients, respectively (**Table 4**). Notably, all the 21 radiological injury cases in the RS and ART group presented as late complications at 3 months after completion of radiotherapy, while the 5 cases in the CCRT group exhibited both early and late radiation damage, and 2 cases had only early radiation damage. The above results indicate comparable quality of life for BCC patients in RS and ART versus CCRT groups at 3 and 6 months after completion of the treatment, and with no evidence of increased the risk of grade ≥ 3 radiological injuries associated with RS+ART.

**Table 3:** Quality of life in patients with BCC receiving different treatments.

	RS and ART (N=384)	CCRT (N=78)	P value
EORTC QLQ-CX24 score, mean ± SD			
3 months after treatment	29.25 ± 4.38	27.00 ± 3.13	0.108
6 months after treatment	22.75 ± 2.77	23.16 ± 2.65	0.689

BCC, bulky cervical cancer; RS and ART, radical surgery and adjuvant radiotherapy; CCRT, concurrent chemoradiotherapy; EORTC QLQ-CX24, Organization for Research and Treatment of Cancer Quality-of-Life questionnaire cervical cancer module; SD, standard deviation.

**Table 4:** Radiation-related complications in patients with BCC receiving different treatments.

RTOG classification of radiation damage	Treatment		Total N (%)	Chi-square value	P value
	RS and ART N (%)	CCRT N (%)			
< grade 3	363 (94.5)	71 (91.0)	434 (93.9)		
≥ grade 3	21 (5.5)	7 (9.0)	28 (6.1)	1.399	0.293
Total, N (%)	384 (83.1)	78 (16.8)			

BCC, bulky cervical cancer; RTOG classification, Toxicity criteria of the Radiation Therapy Oncology Group (RTOG) and the European Organization for Research and Treatment of Cancer (EORTC); RS and ART, radical surgery and adjuvant radiotherapy; CCRT, concurrent chemoradiotherapy.

## Discussion

The prognosis for BCC remains poor, and optimal treatment strategies, particularly for patients without PMI need further exploration. The current clinical assessments exhibited limited accuracy in determining the presence of PMI in BCC. In this study, we retrospectively analyzed the clinicopathological characteristics of 462 BCC patients at our center and found that FIGO stage III-IV and local tumor volume > 16 cm^3^ were independent risk factors for the development of PMI in BCC. Additionally, we investigated the prognostic impacts of PMI in BCC. Although the OS did not differ significantly between the two groups was not statistically significant, patients with PMI exhibited worse PFS than those without PMI. Aside from PMI, FIGO stage III-IV, LVSI, and CCRT increased the risk of tumor recurrence and progression in individuals with BCC. Regarding CSS, tumor diameter, FIGO staging, LVSI, and receipt of various treatments emerged as independent predictors. Given that the optimal treatment of BCC has been a major concern for clinicians, we analyzed the impact of different treatments on prognosis and quality of life in patients with BCC at early stage. In comparison to CCRT, RS and ART improved PFS in those without PMI at FIGO stage I-II, but had no effect on CSS. Additionally, in those with PMI, RS and ART failed to enhance PFS or CSS. No significant difference was found in quality of life between the two groups at 3- and 6- months post-treatment completed. However, there was no significant difference in quality of life between the two groups at 3- and 6-month post-treatment.

The presence or absence of PMI in BCC patients is determined through gynecological examination and pelvic MRI. However, the large local tumor size in BCC often narrows the paracervical space by compression, and the inflammation and edema can lead to thickened paracervical tissue, which complicates PMI assessment by gynecological examination and MRI (21). Yoon et al. (22) included 312 patients with FIGO stage IIB and retrospectively analyzed the consistency of gynecological examination and MRI in determining PMI for UCC patients. Of these individuals, 141 were evaluated with PMI at gynecological examination but without PMI by MRI. All the cases received RH, but only 46 (32.6%) of them had postoperative pathology confirming PMI. 21 (28.4%) of the 74 patients with a clinical diagnosis of FIGO stage IIB who underwent RH in another study revealed postoperative pathologic confirmation of PMI (23). A study by Pan et al. (24), which included 675 UCC patients with tumors ≥ 3cm who underwent RH, indicated that the concordance between MRI and postoperative pathology was 83.2%, and the concordance between physical examination and postoperative pathology was 80.6%. These disparities highlight the need for additional markers to improve PMI assessment in BCC. In the present study, FIGO stage III-IV and tumor volume > 16 cm^3^ were identified as independent risk factors for the development of PMI in BCC, implying an increased risk of PMI in cases with a local tumor volume > 16 cm^3^, invasion of the lower third of the vagina, lymph node metastasis, or distant metastatic disease. A study including 4533 patients with UCC by Li et al. (25) verified that tumor size > 4cm, LVSI, vaginal involvement, pelvic lymph node metastasis, and depth of interstitial infiltration were independent predictors of PMI. While the above findings were partly in line with our findings, their study did not conduct an analysis of the population with tumors larger than 4cm. Our work innovatively incorporated tumor volume for analysis and identified that tumor volume > 16 cm^3^ as an independent prognostic factor for PMI. These results suggest that when PMI is difficult to determine through gynecological examination and pelvic MRI, tumor involvement in the lower 1/3 of the vagina, lymph node metastasis, distant metastasis, and the tumor volume on MRI can aid PMI assessment. Regarding prognosis, our study found that cases with PMI had poorer PFS and OS than those without PMI. However, further univariate and multivariate Cox regression analyses revealed that PMI was an independent risk factor for PFS but not for CSS in patients with BCC. This finding contrasts with previous studies on UCC. A retrospective study investigated 377 UCC patients with FIGO 2018 stage IIIC1 and found that PMI was an independent prognostic factor for their PFS and OS, implying that the prognosis of FIGO IIIC1 patients is hampered by PMI (26). Another monocenter retrospective study from France also confirmed that PMI was a significant factor affecting disease-free survival (DFS), OS in LACC patients undergoing CCRT (27). The present study showed no significant effect of PMI on CSS in BCC patients, which may be attributed to the broader staging range (stages IB3, IIA2, III, and IVA). Moreover, death from other causes likewise impacts OS, whereas our study concentrated on tumor-specific survival, which allowed for a more precise evaluation of PMI’s effect on prognosis. Therefore, PMI assessment is critical to the treatment strategies and prognosis for patients with BCC.

Despite the continuous advancements in radiotherapy and surgical techniques, the treatment of BCC remains a considerable challenge, with poor prognosis and high risks for local recurrence and distant metastasis (13). In our study, after a median follow-up of 65 months, the 5-year survival and CSS rates of BCC patients in our study were 78.2% and 93%, respectively. The CSS rates exceeded the reported 50%-70% range in the literature (13). This discrepancy may be attributed to the large proportion of early-stage patients (77.7% at FIGO stages I and II) enrolled. Our findings revealed that besides FIGO staging and different treatment modalities (RS and ART versus CCRT) were significant factors affecting PFS and CSS in patients with BCC. The study was a single-center retrospective analysis, and most of the participants were at FIGO stage I-II, with fewer at FIGO stage III-IV. We further analyzed the prognosis of the patients at early stage in conjunction with PMI and various treatments. The results indicate that RS and ART can only improve PFS in BCC patients without PMI at early-stage, but have no effect on CSS. Furthermore, RS and ART fail to enhance PFS or CSS in those with PMI. These results align with a retrospective study based on LACC patients at stage IB3 and IIA2 from the SEER database and a single center, which discovered superior outcomes with RS and ART in those with tumors < 6 cm (28). Similarly, another study reported better PFS and OS with RS and ART in patients with local tumors ≥ 4cm and pelvic lymph node (PLN) metastases than with CCRT. However, when the tumors showed PMI, there was no difference in the 7-year OS rate between the two treatment modalities (81.2% vs. 67.4%, P=0.052) (29). These findings suggest that only a subset of BCC patients may benefit from RS and ART, which is in accordance with our investigation. Multiple previous studies have as well explored the effect of neoadjuvant chemotherapy (NACT) combined with RH on the prognosis of BCC. A study by Akhavan S et al. (30) analyzed the outcomes of BCC patients with stage IB3 and IIA2 and demonstrated that those who received NACT with RH exhibited improved OS (3.9 years vs. 3.64 years, P=0.003), and superior DFS (4.5 years vs. 3.6 years, P=0.004) than those who received CCRT. A meta-analysis of 1,259 FIGO 1994-staged IB2, IIA, and IIB patients found no difference in OS (HR 1.08, 95% CI 0.86-1.36) but an increased risk of recurrence (HR 1.32, 95% CI 1.07-1.62) for those treated with NACT and RH compared with those receiving CCRT (31). A phase III multicenter randomized controlled trial (EORTC-55994) in Europe enrolling 626 patients with FIGO 2014 stage IB2-IIB reported no significant differences were recorded between the NACT and RH group and the CCRT group in 5-year OS and PFS rates after a median follow-up of 8.7 years (32). Besides, initial treatment with CCRT is associated with a 30% local control failure rate in BCC patients (33). Although CCRT remains to be the standard treatment for BCC, our findings and the existing literature suggest that a subset of patients, especially those without PMI, may derive greater benefits from RS and ART, which need to be further confirmed with large-scale prospective studies. although CCRT is the standard treatment for BCC. The potential subpopulations gaining benefit require further confirmation with prospective large-sample studies. However, CCRT is still preferred over RS and ART in BCC cases with PMI.

In addition to efficacy, treatment-related adverse effects must be addressed when designing therapeutic regimens for patients with BCC. Our investigation into the impact of various interventions on quality of life revealed no substantial disparities between individuals who received RS and ART and those who received CCRT at the 3- and 6-month post-treatment. In the present study, 9 (2.3%) of the patients treated with RS and ART experienced CD grade 3 surgery-related complications, which is lower than previously reported in the literature (17%), but the incidence of grade ≥ 2 was comparable (34). Besides, no significant difference was observed in grade ≥ 3 radiologic damage between the RS and ART group versus CCRT group, with 21 (5.5%) and 7 (9.0%) cases, respectively. Our study demonstrated that the BCC patients receiving RS and ART did not have an increased risk of grade ≥ 3 radiation injury and maintained comparable quality of life at 3 and 6 months after treatment compared with those undergoing CCRT. CCRT is preferentially recommended for BCC patients due to the large primary tumor, high risks of lymph node metastasis and deep interstitial infiltration, and the need for adjuvant radiotherapy after RH. Yet, this regimen poses combined jeopardies of surgery and radiotherapy to patients, which may compromise their quality of life and survival. In our study, nine cases in the surgery group reported grade ≥3 surgery-related complications, indicating that the surgery group might face dual challenges from surgery and radiotherapy.

The present study identified the factors contributing to PMI in patients with BCC, analyzed the prognostic value of PMI, assessed the effect of different treatments modalities on clinical outcomes and quality of life, and explored the potential populations that could benefit from RS and ART, to provide evidence for clinical decision-making, especially for populations with limited access to radiotherapy, those influenced by socio-cultural preferences, and those preferring surgical treatment. This was a single-center retrospective analysis, a large proportion of participants were at FIGO stage I-II, with fewer at FIGO stage III-IV, which does not fully represent the broader BCC population and thus may result in potential bias. Some factors that may have been overlooked in this analysis, like radiotherapy doses and surgical quality, could potentially introduce bias. Future large-sample, multicenter prospective studies integrating additional factors (e.g., radiotherapy doses and surgical quality) are warranted to corroborate our findings. We also look forward to the results of an ongoing multicenter randomized controlled trial (RCT) by Qiu J et al. (35), which focuses on LACC and may provide further insights.

## Conclusions

The study identified FIGO stage III-IV and tumor volume > 16 cm^3^ as predictors for PMI in patients with BCC, with PMI being associated with poorer prognosis. Compared to those treated with CCRT, individuals at early-stage without PMI who underwent RS and ART demonstrated superior PFS. The RS and ART regimen did not increase the risk of grade ≥ 3 treatment-related adverse events or compromise quality of life. While our findings provide valuable evidence for clinical decision-making in patients with BCC, further large-scale, multicenter prospective studies are expected to evidence our conclusions.

## Data Availability

The original contributions presented in the study are included in the article/supplementary material. Further inquiries can be directed to the corresponding author.
